# Determining Urinary Bile Acid Profiles to Predict Maternal and Neonatal Outcomes in Patients with Intrahepatic Cholestasis of Pregnancy

**DOI:** 10.3390/diagnostics15060657

**Published:** 2025-03-08

**Authors:** Ping You, Min Ding, Xue Li, Yong Shao, Tingting Jiang, Yuanyuan Jia, Yuxuan Wang, Xiaoqing Zhang

**Affiliations:** 1 Key Laboratory of Clinical Laboratory Diagnostics (Ministry of Education of China), School of Laboratory Medicine, Chongqing Medical University, Chongqing 400016, China; youping1989@126.com (P.Y.); dingmin@cqmu.edu.cn (M.D.); lixue99lx@163.com (X.L.); 2022110517@stu.cqmu.edu.cn (T.J.); 2023111501@stu.cqmu.edu.cn (Y.J.); w2023221636@163.com (Y.W.); 2 Department of Obstetrics and Gynecology, The First Affiliated Hospital of Chongqing Medical University, Chongqing 400016, China; cqshaoyong@163.com

**Keywords:** adverse perinatal outcomes, urinary BAs, bile acid profiles, intrahepatic cholestasis of pregnancy

## Abstract

**Objective**: Intrahepatic cholestasis of pregnancy (ICP) is associated with an elevated risk of adverse perinatal outcomes, including perinatal morbidity and mortality. The objectives of this study were to evaluate the bile acid (BA) metabolism profiles in the urine of patients with ICP and to investigate the association between specific BAs and maternal and neonatal outcomes in patients with ICP. **Methods:** A total of 127 Chinese women with ICP and 55 healthy pregnant women were enrolled in our retrospective study. Spot urine samples and clinical data were collected from pregnant women from January 2019 to December 2022 at the First Affiliated Hospital of Chongqing Medical University, Chongqing. Based on total bile acid (TBA) levels, the ICP group was subdivided into mild (10–40 μmol/L) and severe (≥40 μmol/L) ICP groups. Patients in the ICP group were further divided into two categories according to neonatal outcomes: an ICP with adverse pregnancy outcomes group and an ICP with non-adverse pregnancy outcomes group. Metabolites from maternal urine were collected and analyzed using ultra-high-performance liquid chromatography–triple quadrupole time-of-flight mass spectroscopy (UPLC-triple TOF-MS). **Results:** Significant differences were observed between the mild and severe ICP groups in the onset time of symptoms, gestational weeks at time of ICP diagnosis, the duration of using ursodeoxycholic acid (UDCA) drugs during pregnancy, gestational age at delivery, premature delivery, and cesarean delivery. The expression levels of the composition of different urinary bile acids including THCA, TCA, T-ω-MCA, TCA-3-S, TCDCA-3-S, TDCA-3-S, GCDCA-3-S, DCA-3-G and GDCA-3-G were remarkably higher in the ICP with adverse pregnancy outcomes group than those in the ICP with non-adverse pregnancy outcomes group and the control group. The single-parameter model used to predict adverse pregnancy outcomes in ICP had similar areas under the curve (AUCs) of the receiver operating characteristic (ROC), ranging from 0.755 to 0.869. However, an AUC of 0.886 and 95% CI were obtained by the index of combined urinary bile acids in multiple prediction models (95% CI 0.790 to 0.983, *p* < 0.05). TCA-3-S in the urinary bile acids had a strong positive correlation with the aspartate aminotransferase (AST) level (*r* = 0.617, *p* < 0.05). Furthermore, TCDCA-3-S and GCDCA-3-S in the urinary bile acids had a strong positive correlation with the alanine aminotransferase (ALT) level (*r* = 0.607, *p* < 0.05; *r* = 0.611, *p* < 0.05) and AST level (*r* = 0.629, *p* < 0.05; *r* = 0.619, *p* < 0.05). **Conclusions:** Maternal urinary bile acid profiles were prominent for the prognosis of maternal and neonatal outcomes of ICP. Elevated levels of TCA-3-S, TCDCA-3-S, and GCDCA-3-S in urine might be important predictors for indicating adverse pregnancy outcomes in ICP.

## 1. Introduction

Intrahepatic cholestasis of pregnancy (ICP) is an idiopathic pregnancy-related disorder characterized by maternal pruritus, jaundice, elevated hepatic aminotransferase activity, and elevated serum total bile acid (TBA) concentrations. Patients generally recover after delivery, and thus it is considered a benign condition in pregnant women [1]. However, ICP is significantly associated with an increased risk of meconium-stained amniotic fluid (MSAF), spontaneous and iatrogenic preterm birth (PTB), respiratory distress syndrome, neonatal intensive care unit (NICU) admission, intrauterine growth retardation, fetal distress or asphyxia, and even stillbirth [2,3]. Consequently, the management of ICP still remains a significant challenge for obstetricians, given the elevated prevalence of premature rupture of membranes and postpartum hemorrhages and the increased risk of adverse neonatal outcomes [2,4].

Among the known symptoms of ICP, high levels of TBA in particular are associated with an elevated risk of perinatal morbidity and mortality [5,6,7]. A maternal serum TBA level of more than 10 μmol/L is used as a cut-off for the diagnosis of ICP. Furthermore, the incidence of adverse pregnancy outcomes has been found to increase in conjunction with elevated TBA levels. It has been demonstrated that a maternal TBA level of more than 40 μmol/L is indicative of an increased risk of fetal complications [8]. Moreover, each 1 μmol/L increase in TBA levels is associated with a 1% to 2% rise in the likelihood of fetal complications. In addition, TBA levels of more than 100 μmol/L increase the risk of stillbirth [9].

Bile acid (BA) metabolic profiling has shown that cholic acid (CA), chenodeoxycholic acid (CDCA), and their glycine (G)- and taurine (T)-conjugated forms predominate in the TBA profiles of patients with ICP. As the metabolism of BAs changes during pregnancy, it is necessary to understand the differences in the characteristics of BAs in ICP patients, which is key to understanding why different types of ICP lead to different rates of adverse pregnancy outcomes [10]. However, studies investigating the relationship between specific BAs and the incidence of perinatal complications—particularly for preterm births, which are the most common outcome of ICP—are still lacking.

In this study, we evaluated the characteristics of BA metabolism profiles in urine, an easily accessible biofluid and a non-invasive source of metabolites, from patients with ICP using ultra-high-performance liquid chromatography–triple quadrupole time-of-flight mass spectroscopy (UPLC-triple TOF-MS/MS). In addition, we investigated whether changes in individual BAs were associated with the incidence of ICP with adverse pregnancy outcomes. This study may contribute to developing convenient sampling methods and identifying potential biomarkers for early detection of and intervention in ICP in clinical practice.

## 2. Methods

A total of 127 Chinese women with ICP and 55 cases of healthy pregnant women were enrolled in our retrospective study. Spot urine samples and clinical data were collected from pregnant women from January 2019 to December 2022 at the First Affiliated Hospital of Chongqing Medical University, Chongqing. Written informed consent was obtained from all subjects, and all experimental protocols were approved by the ethics committee of the First Affiliated Hospital of Chongqing Medical University (No: 2019-166). ICP was diagnosed according to the ICP guidelines from the Royal College of Obstetricians and Gynecologists edition II [11]. The inclusion criteria for ICP were as follows: participants had to have a fasting serum TBA level of 10 µmol/L or more, or a postprandial serum TBA level of 19 µmol/L or more, with or without other unexplained liver abnormalities. The exclusion criteria were as follows: individuals who had in vitro fertilization and embryo transfer [3]; elevated liver enzymes and low platelet syndrome; preeclampsia; specific drug users (i.e., paracetamol or morphine); and autoimmune diseases and other hepatic diseases and diseases affecting liver function tests. UDCA treatment was used for patients diagnosed with ICP [12]. The conventional drug treatment regimen for patients with ICP was oral UDCA 10 ~ 15 mg·kg^−1^·d^−1^, administered twice or three times daily. The control group comprised only those healthy pregnant women matched with cases on the basis of age and weeks of gestation during the same period. Women with a history of gallstones or cholecystopathy, pruritus, drug consumption, hepatitis, or any other diseases damaging hepatobiliary function were excluded from the control group. The patients’ clinical data are shown in Table 1. Urine samples were obtained from patients in a random state at the primary diagnosis of ICP. Mid-stream random urine samples were collected and stored at −80 °C until further analysis.

**Figure 1:**
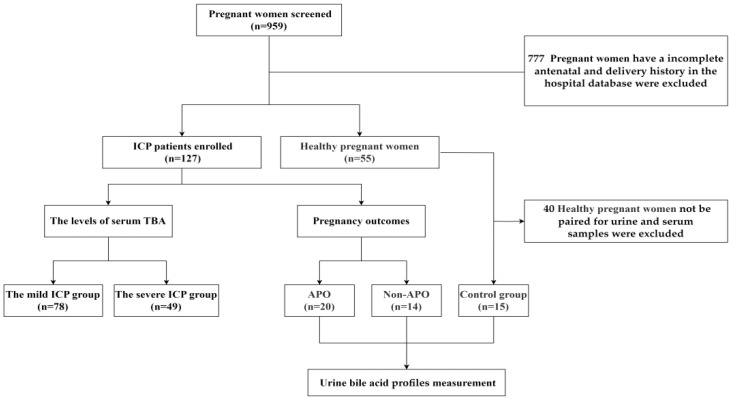
A flowchart of enrolled and analyzed samples in this study.

Microsoft Excel (version 16.61) was utilized as a random number generator to facilitate data collection and sampling in the control group. In the study, a total of 959 pregnant women were investigated. Pregnant women with ICP who did not have a complete antenatal and delivery history in the hospital database were excluded, as well as pregnant women with ICP who could not complete the pairing of urine and serum samples. In the control group, pregnant women from the same population and year as the case group were selected from the hospital database and matched for age, gestational age, and maternal history to exclude those with incomplete antenatal and delivery records in the hospital’s electronic database and those who could not be matched for urine and serum samples. Consequently, 777 pregnant women were excluded from the study. A total of 182 pregnant women according to the inclusion criteria were enrolled for the experiment, including a control group (*n* = 55) and case group (*n* = 127). Urinary bile acid profiles were performed in pregnant women with adverse pregnancy outcomes (APOs) (*n* = 20), pregnant women with non-APOs (*n* = 14), and the control group (*n* = 15), as shown in Figure 1.

### 2.1. Maternal Variables

Peak TBA levels were used in to classify the severity of ICP. Patients with a TBA level ≥ 10 μmol/L were divided into two different severities of ICP according to the maximum TBA level throughout the entire pregnancy. The specific criteria for the mild and severe ICP groups are as follows [11]. (1) Mild ICP: peak TBA levels from 10 μmol/L to 40 μmol/L; (2) severe ICP: peak TBA levels > 40 μmol/L.

### 2.2. Neonatal Outcomes

ICP patients, using perinatal outcomes including abortion, PTB (gestational age less than 37 weeks), fetal heart rate at delivery, birth length, birth weight, NICU admission, MSAF, Apgar score, and gestational age, were divided into two subtypes (the ICP group with adverse pregnancy outcomes and the ICP group with non-adverse pregnancy outcomes). According to the ultrasound measurements: estimation of gestational week by parameters such as gestational sac diameter (GS); parietal rump length (CRL) in early pregnancy (<14 weeks) or biparietal diameter (BPD); and femur length (FL). The gestational age was then calculated utilizing a specified formula. The fetal heart rate (110–160 beats per minute) was typically monitored continuously throughout the course of labour by means of electronic fetal monitoring (EFM). The birth length (46–54 cm) of the newborn was measured using an infantometer, ensuring that the newborn was lying flat on their back with the head and feet in a horizontal position. The birth weight (2500–4000 g) was undertaken using an electronic baby scale, with the newborn in a supine position and the scale surface maintained in a clean condition. MSAF was typically observed in the 37th week of gestation and beyond, with its prevalence being higher in second-trimester pregnancies (>42 weeks). Studies have demonstrated that the prevalence of MSAF in the Chinese population ranges from 5% to 15%, with variations across different regions and population groups.

### 2.3. Urinary Bile Acid Profile Measurement

Quantitative and semi-quantitative analysis of bile acid profiles in spot urine were performed by using a pseudo-targeted analysis and isotope labeling strategy based on the ultra-high-performance liquid chromatography–triple quadrupole time-of-flight mass spectroscopy (UPLC-triple TOF-MS/MS) platform [13]. An ExionLCTM UPLC system (Shimadzu, Kyoto, Japan) coupled with a Triple TOF MS 6600+ (AB Sciex, Singapore) with an electrospray ionization (ESI) system was used for the analysis. The analytical conditions for the chromatography and mass spectrometry referenced our previous studies [13]. The creatinine level was used to correct for the urine volume.

### 2.4. Statistical Analysis

SPSS 25.0 (2017, IBM Corporation, Armonk, NY, USA) and GraphPad Prism 9 (2020, San Diego, CA, USA) were utilized for statistical analysis and figure presentation. Statistical differences for the three groups were evaluated by one-way analysis of variance (ANOVA) or the Kruskal–Wallis test. Pearson’s correlation or Spearman’s correlation was used to analyze the correlation between the sequencing data and experimental results and the correlation between the experimental results and clinical data. The receiver operating characteristic (ROC) curve analysis was performed to assess the diagnostic value of the bile acid biomarkers for ICP. Sensitivities and specificities (with a 95% confidence interval [CI]) were calculated based on the area under the curve (AUC). An AUC of more than 0.7 is regarded as an acceptable level for discrimination. Binary logistic regression analysis was adopted to produce ROC curves for the diagnostic and predictive utility of maternal urine biomarker levels. All data are presented as means ± standard errors (SEs) and median and interquartile range. For all comments, when the *p* value was less than 0.05, it was considered statistically significant and labelled with * *p* < 0.05, ** *p* < 0.01, and *** *p* < 0.001.

## 3. Results

### 3.1. Clinical Features of the Pregnant Women in Different Groups

In total, 127 pregnant women with ICP met the eligibility criteria. The average age was 30.22 years (ranging from 25 to 41 years old). Thirteen patients had a history of ICP. Moreover, 54 (42.51%) patients had clinical symptoms. Among the three groups, no significant differences were observed in maternal ages, weight gain in pregnancy, or multipara. In addition, no significant differences in multipara, history of ICP, skin pruritus, or the number of patients treated with UDCA during pregnancy (all *p* >  0.05) were observed between the mild (10–40 μmol/L) and severe ICP groups (>40 μmol/L). However, significant differences were observed in the time of symptom onset, gestational weeks at the time of ICP diagnosis, the duration of UDCA drug use during pregnancy, the peak level of TBA (before and after delivery), the peak level of TBA (before and after treatment with UDCA), gestational age at the peak level of TBA (before treatment with UDCA), gestational age at delivery, and premature and cesarean deliveries between the two groups (all *p* <  0.05). Compared with the control group, pregnant women with ICP were more likely to have a preterm labor, and the severe ICP group (>40 μmol/L) had a higher rate of cesarean delivery, as shown in Table 1.

### 3.2. Biochemistry Function Tests in the Controls, Mild, and Severe ICP Pregnant Women

ALT and AST were found to be independent risk factors for adverse perinatal outcomes in patients with ICP [14]. Therefore, serum biochemical and clinical indicators were investigated between the mild and severe ICP pregnant women. No significant differences in the serum biochemical and clinical indicators at the time of the diagnosis of ICP or at the time of delivery were observed between the two groups (all *p* >  0.05), as shown in Table 2.

### 3.3. Perinatal Outcomes in the Control Group and Different Types of ICP Groups

No significant differences were observed in the risk of abortion, PPH, 24 h blood loss, macrosomia, Apgar score < 7 at 5 min, and meconium-stained amniotic fluid (MSAF) (all *p* >  0.05) between the mild ICP group and the severe ICP group, as shown in Table 3. However, significant differences were observed in the risk of iPTB, NICU transfer, low birth weight, and birth weight or length (all *p* <  0.05). The incidence of these perinatal complications was substantially higher in the severe ICP group than in the mild ICP group and the control group (all *p* <  0.001). In the severe ICP group, the incidence of adverse pregnancy outcomes was as high as 69.39%, a statistically significant difference when compared to that of the control group and the mild ICP group (*p* <  0.001). For the severe ICP group in our study, the incidence of PTB, NICU admission, and low birth weight was found to be 53.06% (26 of 49), 53.06% (26 of 49), and 16.33 (8 of 49), respectively. The mean birth weights of infants in the mild ICP and severe ICP group were 3100 g (1940–4450) and 2845 g (1420–4100) (*p* <  0.05), respectively. The mean birth lengths of infants in the mild ICP and severe ICP group were 49 cm (44–54), and 48 cm (37–51) (*p* <  0.001), respectively. These results indicate that the peak TBA level was closely correlated with adverse pregnancy outcomes.

### 3.4. Clinical Information and Urinary Bile Acid Metabolism Characteristics

Changes in clinical information and urinary bile acid metabolism characteristics with the progression of pregnancy were investigated in pregnant women without ICP, ICP patients with non-adverse pregnancy outcomes, and ICP patients with adverse pregnancy outcomes, as shown in Table 4. The results revealed that hepatic functions except for pre-albumin (PA) and total protein (TP) varied among the three groups. Globulin (GLB), ALT, AST, total bilirubin (TB), direct bilirubin (DB), total bile acid (TBA), alkaline phosphatase (ALP), and gamma-glutamyl transpeptidase (GGT) were elevated in the ICP with adverse pregnancy outcomes group (all *p* <  0.05). A total of 40% of this group had a TBA level higher than 40  μmol/L, and the differences were statistically significant when compared to the control group and the ICP with non-adverse pregnancy outcomes group. Significant differences were observed in gestational age at delivery, premature delivery, cesarean delivery, fetal heart rate at delivery, birth weight, NICU transfer, MSAF, and Apgar score < 7 at 5 min among the three groups (all *p* <  0.05).

**Figure 2:**
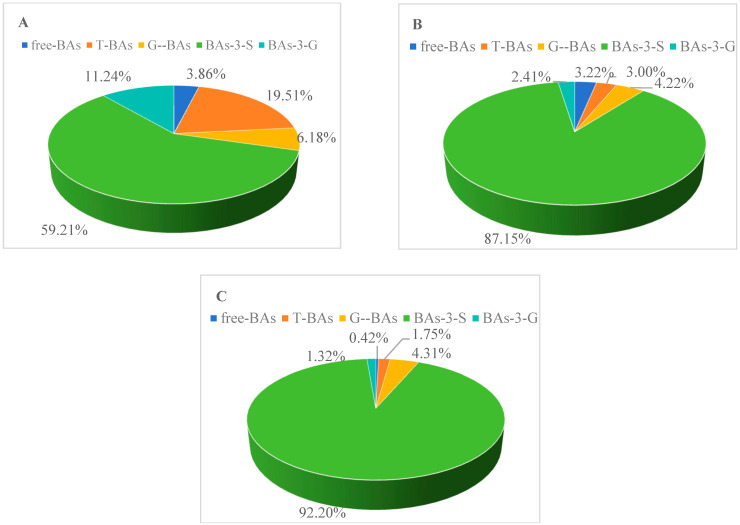
The composition of bile acids in different groups. (**A**) Control group; (**B**) ICP with non-adverse pregnancy outcomes group; (**C**) ICP with adverse pregnancy outcomes group.

**Figure 3:**
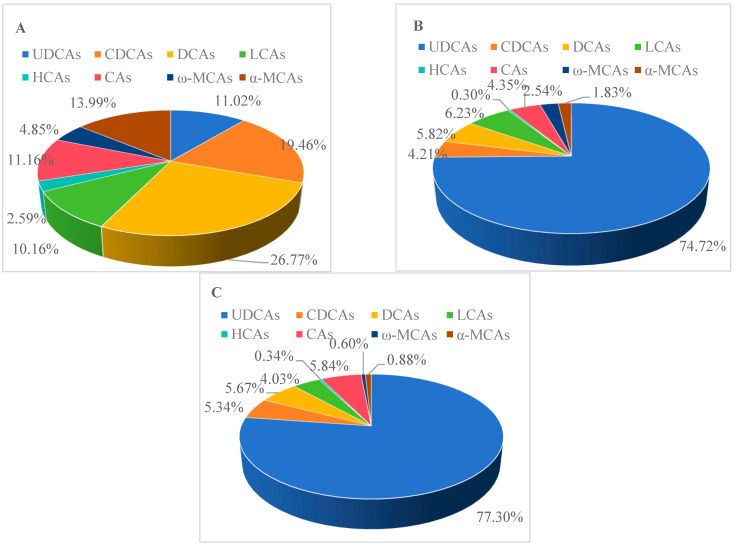
The composition of different bile acids in different groups. (**A**) Control group; (**B**) ICP with non-adverse pregnancy outcomes group; (**C**) ICP with adverse pregnancy outcomes group.

To further understand the urinary BA metabolism profiles of different types of ICP patients, the BA profiles in pregnant women without ICP were examined via UPLC-triple TOF-MS. Although TBA levels remained constant throughout the second and third trimesters, BA profiles changed, as shown in Figure 2. The BA pool, free BAs, T-BAs, G-BAs, BAs-3-S and BAs-3-G accounted for 0.42%, 1.75%, 4.31%, 92.20% and 1.32%, respectively, of the total in the ICP with adverse pregnancy outcomes group (Figure 3). However, UDCAs, CDCAs, DCAs, LCAs, HCAs, CAs, ω-MCAs, and α-MCAs accounted for 77.30%, 5.34%, 5.67%, 4.03%, 0.34%, 5.84%, 0.60%, and 0.88%, respectively, in the ICP with adverse pregnancy outcomes group.

**Figure 4:**
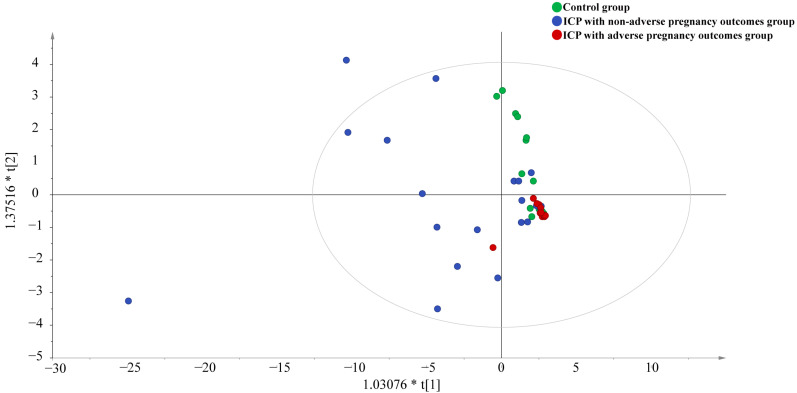
Score plots of OPLS-DA model for bile acid profiling in the ICP with adverse pregnancy outcomes group, ICP with non-adverse pregnancy outcomes group, and the control group. * means multiplication sign.

An orthogonal projection to latent structure discriminant analysis (OPLS-DA) of bile acid metabolic profiles was performed based on 38 bile acids found in the urine from the ICP with adverse pregnancy outcomes group, ICP with non-adverse pregnancy outcomes group, and the control group, as shown in Figure 4. The results showed a trend toward separation of the three groups, with significant differences in bile acid metabolic profiles. More significant differences appeared in individuals in the ICP with adverse pregnancy outcomes group. The explanatory rate of the model R^2^X was 0.814, and the predictive rate Q^2^ was 0.476.

**Figure 5:**
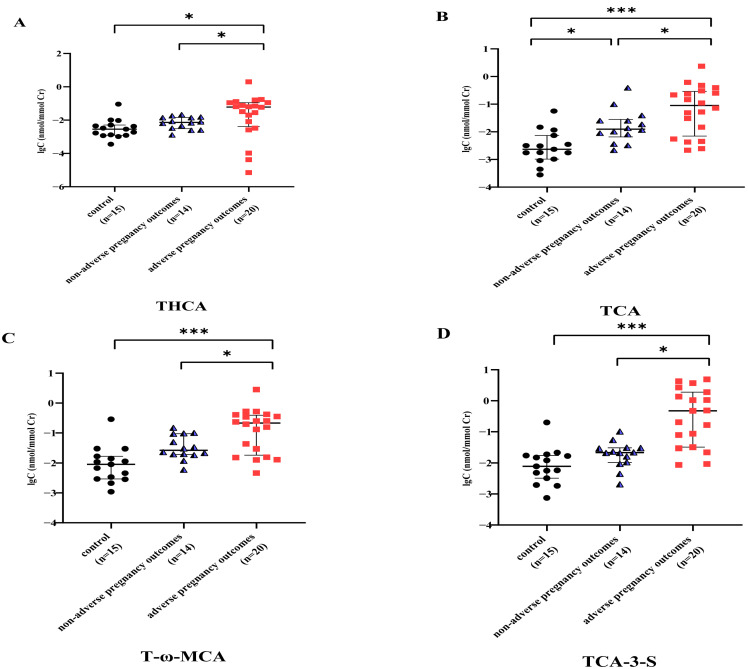

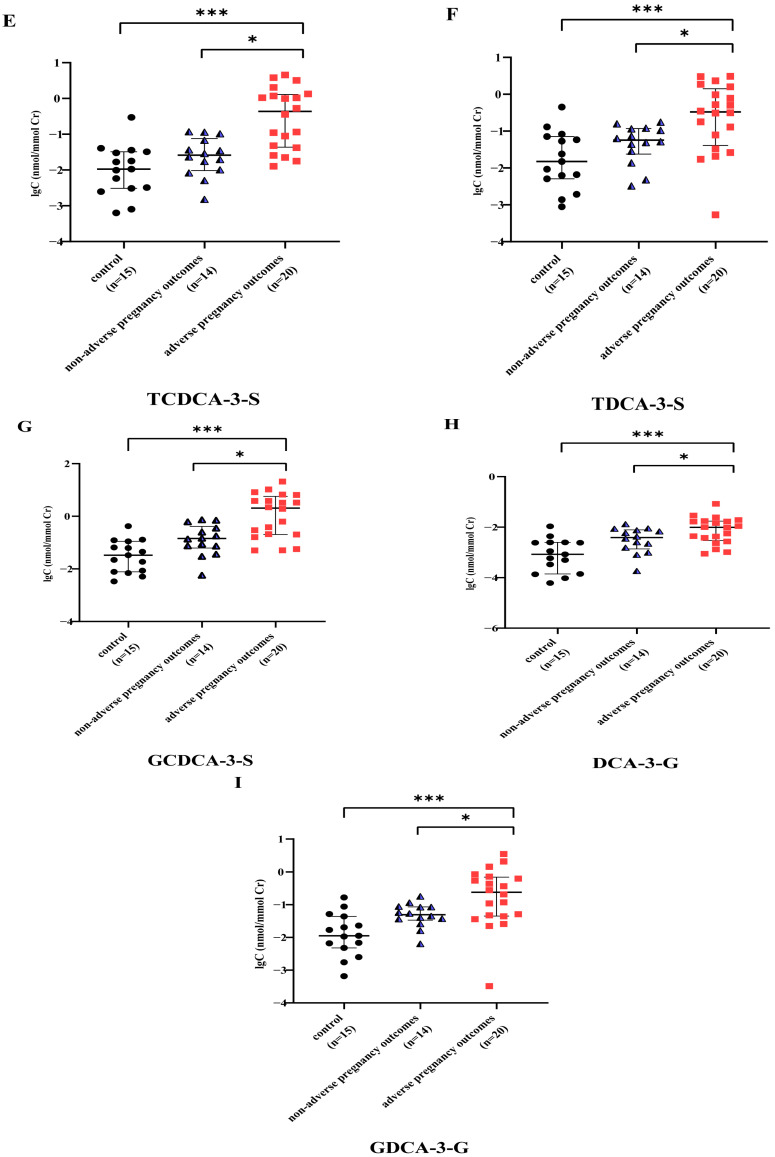
Validation of alteration in biomarker levels (nmol/mmol Cr) in the ICP with the adverse pregnancy outcomes group. (**A**) Levels of THCA in the urine; (**B**) levels of TCA in the urine; (**C**) levels of T-ω-MCA in the urine; (**D**) levels of TCA-3-S in the urine; (**E**) levels of TCDCA-3-S in the urine; (**F**) levels of TDCA-3-S in the urine; (**G**) levels of GCDCA-3-S in the urine; (**H**) levels of DCA-3-G in the urine; (**I**) levels of GDCA-3-G in the urine (* means *p* value < 0.05, *** means *p* value < 0.001).

Specifically, levels of THCA, TCA, T-ω-MCA, TCA-3-S, TCDCA-3-S, TDCA-3-S, GCDCA-3-S, DCA-3-G, and GDCA-3-G predominated in urinary BA pools (all *p* < 0.05) in the ICP with adverse pregnancy outcomes group, as shown in Table 5. Nevertheless, compared with the healthy pregnant women and patients with ICP with non-adverse pregnancy outcomes, this group had higher levels of THCA, TCA, T-ω-MCA, TCA-3-S, TCDCA-3-S, TDCA-3-S, GCDCA-3-S, DCA-3-G, and GDCA-3-G in their urine. These differences resulted in a significant effect in the ICP with adverse pregnancy outcomes group, as shown in Figure 5.

### 3.5. Predictive Values of Urinary Bile Acid Levels for Adverse Pregnancy Outcomes in ICP

**Figure 6:**
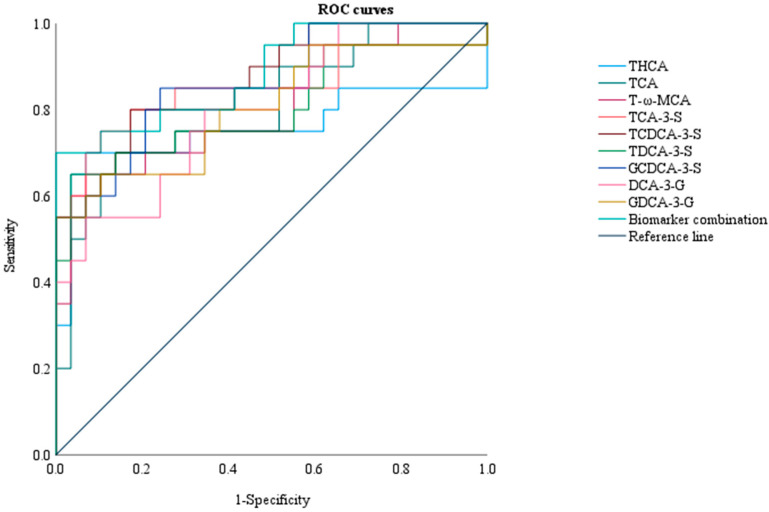
Diagnostic and predictive utility of maternal urine biomarker levels (nmol/mmol Cr) in ICP with adverse pregnancy outcomes.

Logistic regression revealed that elevated TBA, THCA, TCA, T-ω-MCA, TCA-3-S, TCDCA-3-S, TDCA-3-S, GCDCA-3-S, DCA-3-G, and GDCA-3-G levels were risk factors for ICP with adverse pregnancy outcomes, as shown in Table 6. The single-parameter models used to predict ICP with adverse pregnancy outcomes had similar areas under the curve (AUCs), as shown in Figure 6. Moreover, the combination of the above BAs improved the AUC of 0.886 and 95% CI in multiple prediction models (95% CI 0.790 to 0.983, *p* < 0.05), indicating that it could be a potential index for the prediction of ICP with adverse pregnancy outcomes.

### 3.6. Correlation of Urinary Bile Acid Metabolism Characteristics with Clinical Biochemical Indicators

**Figure 7:**
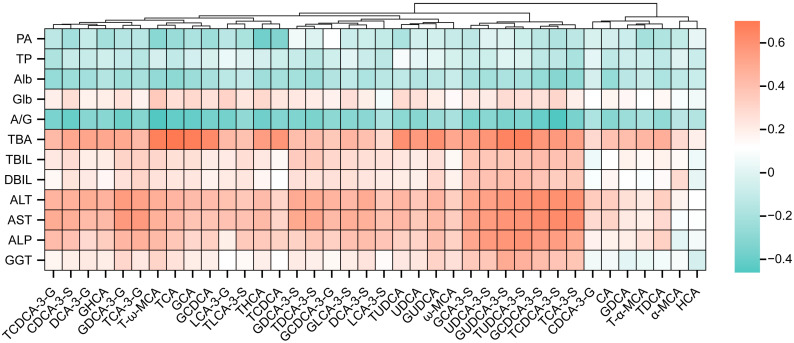
Heatmap of the Spearman correlation coefficient between the urinary bile acids and clinical indices.

Spearman correlation analysis was used to explore the correlation between urinary bile acids and serum biochemical indicators. The results showed that UDCA-3-S, TCA-3-S, TUDCA-3-S, TCDCA-3-S, GCA-3-S, GUDCA-3-S, and GCDCA-3-S all had strong positive correlations with ALT, AST, TBIL, DBIL, and GGT, suggesting that these urinary bile acids might be related to liver injury, as shown in Figure 7. The TCA-3-S in the urinary bile acid metabolism had a strong positive correlation with AST (*r* = 0.617, *p* < 0.05). Moreover, TCDCA-3-S and GCDCA-3-S had strong positive correlations with ALT (r = 0.607, *p* < 0.05 and r = 0.611, *p* < 0.05, respectively) and AST (r = 0.629, *p* < 0.05 and r = 0.619, *p* < 0.05, respectively), as shown in Table 7.

## 4. Discussion

ICP is the most common pregnancy-related liver disease [15]; the aetiology of ICP may be multifactorial, involving genetic, hormonal, and environmental factors. Physical activity and a nutritionally balanced diet may improve the symptoms or reduce the incidence of ICP. At present, UDCA is the drug of choice for the treatment of ICP in first-line settings [16,17,18,19]. ICP has been reported to increase the incidence of perinatal complications, including meconium-stained amniotic fluid (MSAF), spontaneous and iatrogenic preterm birth (PTB), respiratory distress syndrome, neonatal intensive care unit (NICU) admission, and even intrauterine fetal death [10,20,21,22,23,24].

Currently, the clinical classification criteria for ICP are mainly based on serum TBA levels [25]. Here, the critical point for the diagnosis of ICP was considered a TBA level of more than 10 µmol/L. Patients with mild ICP were defined as those with TBA levels from 10 µmol/L to 40 µmol/L, and patients with severe ICP were defined as those with TBA levels of more than 40 µmol/L (as suggested in the literature) [26,27]. This may impede the early diagnosis of ICP, as serum TBA levels are unable to differentiate between ICP patients with low levels of pruritus and pregnant women without ICP. Furthermore, a consensus on the diagnostic threshold for liver enzymes has not yet been reached [7]. The results of our study demonstrated that the severe ICP group exhibited symptoms of cholestasis at an earlier stage and thus were diagnosed at an earlier stage. Before treatment with UDCA, the severe ICP group reached a peak level of TBA, and treatment with UDCA lasted for a longer period of time than in the mild ICP group. The prevailing medical consensus on the timing and mode of delivery for ICP patients is to recommend delivery between 37–38 weeks of gestation in order to reduce the risk of adverse fetal outcomes. The precise timing must be assessed on a case-by-case basis, taking into account the patient’s clinical presentation, bile acid level, and fetal status [11,12]. In this study, the incidence of adverse pregnancy outcomes was higher in the severe ICP group than in the mild ICP group, including premature birth (PTB), high fetal heart rates at birth, low birth weights, short body lengths, and low birth weights (LBW) for the fetuses. Additionally, an elevated rate of neonatal intensive care unit (NICU) admissions was also observed in the severe ICP group compared to that in the mild ICP group. Nevertheless, statistically insignificant differences were observed between the two groups in terms of the number of hemorrhages at the time of delivery and 24 h bleeding, incidence of macrosomia, MSAF, and Apgar scores < 7 at 5 min.

Bile acids are classified into two categories: primary and secondary. Primary bile acids are derived from cholesterol in the liver and undergo a series of reactions that result in the formation of CA and CDCA. These are then conjugated with glycine and taurine to form GCA, GCDCA, TCA, and TDCA. Bile acids can be further classified as hydrophilic or hydrophobic. Hydrophobic bile acids act as scavengers by dissolving cell membrane lipids and increasing cell membrane permeability, which ultimately results in hepatocyte necrosis. Hydrophilic bile acids (e.g., UDCA) facilitate the metabolism of hydrophobic bile acids and modify the composition of bile salts by increasing bile acid and phospholipid contents. This protects hepatocyte membranes and acts as a choleretic agent against the toxicity of hydrophobic bile acids. The most common types of free and conjugated bile acids in the human body in order of hydrophilicity are as follows: UDCA > CA > CDCA > DCA > LCA, taurine-conjugated bile acids > glycine-conjugated bile acids. Free bile acids are the least common [28,29].

The sulphation process, catalyzed by sulphotransferase 2A1, increases the solubility of BAs, facilitating their excretion in urine and feces. Furthermore, this process reduces the toxicity of these compounds [30]. In humans, urinary BAs are primarily sulphated. Total urinary SBAs were found to be significantly elevated in ICP patients, with a notable increase observed for glycine-amidated and taurine-amidated sulphated BAs. These findings indicate that sulphation might be the main pathway for the elimination of BAs from the body [31]. Additionally, urinary BA sulphates demonstrated superior selectivity and specificity for predicting intrahepatic cholestasis during pregnancy compared to serum or urinary total BAs [32,33].

Although the risk of ICP to the mother is minimal, it is closely associated with poor perinatal outcomes, including preterm labor and stillbirth [7]. Preterm labor is one of the most common clinical complications of ICP [34]. In patients exhibiting clinical signs of ICP for whom a definitive diagnosis cannot be established, a combination of tests should be employed to minimize the incidence of adverse pregnancy outcomes [26]. A powerful platform for the discovery of new diagnostic biomarkers is provided by new metabolomics technology based on UPLC-triple TOF-MS. Urine is an important biological sample in metabolomic studies because of its non-invasive collection and volume illimitation. In addition, metabolic dysregulation can be discovered from urine samples. Thus, urine samples can provide insights into system-wide changes in response to physiological challenges or disease processes. However, urine samples can be diluted, which means that the analytes are variable. In this study, creatine was used as an internal standard to calibrate changes in urine volume. This makes urine an excellent biological sample for metabolomic studies [35]. The aims of this study were to investigate the metabolic profile of urinary BAs in ICP patients during pregnancy and to identify potential biochemical markers for the prediction of adverse pregnancy outcomes. The ICP with adverse pregnancy outcomes group had an increased rate of cesarean sections, elevated adverse fetal outcomes, and were prone to iPTB, low birth weights, increased NICU admissions, MSAF and Apgar scores < 7 at 5 min compared with the ICP with non-adverse pregnancy outcomes group and the control group.

Urinary BA metabolic profiles changed significantly in the ICP with adverse pregnancy outcomes group compared with the ICP with non-adverse pregnancy outcomes group and the control groups, and the compositions of different types of bile acids were different between the two groups. The THCA, TCA, T-ω-MCA, TCA-3-S, TCDCA-3-S, TDCA-3-S, GCDCA-3-S, DCA-3-G, and GDCA-3-G levels dominated the urinary BA pool in the ICP with adverse pregnancy outcomes group, exhibiting statistically significant differences. Maternal urinary biomarker levels (nmol/mmol Cr) had an important role in the diagnosis and prediction of adverse pregnancy outcomes in ICP, with PTB having diagnostic value and NICU admission, MSAF, and Apgar scores of < 7 at 5 min having predictive value. The combination of the above nine biomarkers led to the maximum AUC of 0.886.

Most current studies support the involvement of inflammatory mediators in the development of ICP and that bile acid stasis directly activates inflammatory pathways in the liver, favoring the initiation of an inflammatory response and the accumulation of pro-inflammatory mediators that might lead to liver damage [36]. Consequently, ICP is often associated with inflammatory factors and abnormal liver enzyme levels to varying degrees [37]. In addition, normal liver function is essential for maintaining a normal pregnancy [38]. As pregnancy progresses, the liver progressively increases in volume to meet the nutritional and metabolic demands of fetal and maternal growth and development; this is accompanied by elevated biochemical markers that represent the compensatory function of the liver during pregnancy [10,39,40]. As demonstrated by liver function tests, the ICP with adverse pregnancy outcomes group had lower ALB levels than the non-adverse pregnancy outcomes group and the control groups, whereas TBA, TBIL, ALT, AST, ALP and GGT levels were statistically significantly increased in the ICP with adverse pregnancy outcomes group. The levels of THCA, TCA, T-ω-MCA, TCA-3-S, TCDCA-3-S, TDCA-3-S, GCDCA-3-S, DCA-3-G, and GDCA-3-G predominated in the urinary BA pools in the ICP with adverse pregnancy outcomes group, in contrast to the control group and ICP with non-adverse pregnancy outcomes groups. Moreover, UDCA-3-S, TCA-3-S, TUDCA-3-S, TCDCA-3-S, GCA-3-S, GUDCA-3-S, and GCDCA-3-S all had strong positive correlations with ALT, AST, TBIL, DBIL, and GGT.

In particular, the urinary bile acid profiles of TCA-3-S, TCDCA-3-S, and GCDCA-3-S were found to be statistically different between the non-adverse pregnancy outcomes group and the control group for predicting adverse pregnancy outcomes of ICP patients. Furthermore, a statistically significant correlation was observed between these profiles and the liver enzymes of ALT and AST. A combined analysis of urinary bile acid profiles and hepatic function improved the diagnostic precision of adverse pregnancy outcomes in ICP.

## 5. Strengths and Limitations

This study was conducted in a tertiary-A general hospital in southwestern China and followed strict procedures. Furthermore, the study design was a retrospective cross-sectional analysis. Therefore, the results of the naturalistic study reflected the pregnancy and foetal outcomes of ICP patients in this region well. The urinary bile acid profiles offered a convenient sampling method for bile acid monitoring during pregnancy in patients with ICP. Furthermore, it provided clinicians with a new approach to preventing adverse pregnancy outcomes in ICP.

However, this study was conducted over a relatively brief period, and the sample size of the participants was limited. Moreover, the non-randomised sampling study was not representative of all pregnant women with ICP. In addition, given the limited number of samples studied herein, further investigations will be necessary to confirm these observations through targeted metabolomics technology using a larger sample size.

## 6. Conclusions

In conclusion, our findings indicate that patients with ICP who experienced adverse pregnancy outcomes exhibited distinctive urinary bile acid (BA) profiles. In addition, the metabolic changes in urinary BAs were revealed, which may contribute to elucidating the potential mechanisms underlying the occurrence and development of ICP. The reduction in ALB levels and the increases in TBA, TBIL, ALT, AST, ALP, and GGT levels indicated an elevated risk of adverse pregnancy outcomes associated with ICP. This suggests that a high hepatic load might be a contributing factor to the increased risk of adverse pregnancy outcomes. Furthermore, elevated percentages of TCA-3-S, TCDCA-3-S, and GCDCA-3-S in urine might be indicative of adverse pregnancy outcomes in patients with ICP.

## Figures and Tables

**Table 1:** Characteristics of the women included in the study.

Variables	Control Group (*n* = 55)	The Mild ICP Group (*n* = 78)	The Severe ICP Group (*n* = 49)	*p* Value
Maternal age, mean (range), years	30 (22–41)	30 (26–41)	30 (25–35)	0.784
Maternal pre-pregnancy BMI, mean (range), (kg/m^2^)	21.41 (17.26–28.93)	21.80 (16.41–31.11)	21.00 (17.19–29.30)	0.590
Maternal BMI at delivery, mean (range), (kg/m^2^)	26.32 (19.65–37.81)	26.75 (20.37–35.06)	25.78 (19.63–31.44)	0.428
Weight gain in pregnancy, mean (range), kg	13.87 (3.50–25.50)	13.01 (0–30.00)	11.51 (1.00–28.60)	0.097
Multipara, *n* (%)	14 (25.45)	18 (23.08)	16 (32.65)	0.594
Cesarean delivery, *n* (%)	19 (34.55)	55 (70.51) *	42 (85.71) *^,#^	<0.001
Gestational age at delivery, mean (range), (weeks)	39.03 (35.28–41.28)	38.05 (34.00–40.43) *	36.10 (32.14–40.00) *^,#^	<0.001
Gestational age at delivery <37 wk, *n* (%)	3 (5.45)	8 (10.26)	27 (55.10) *^,#^	<0.001
Twin or multiple pregnancy, *n* (%)	0 (0)	6 (7.69)	2 (4.08)	0.417
History of ICP, *n* (%)	-	8 (10.26)	5 (10.20)	0.992
Skin pruritus, *n* (%)	-	31 (39.74)	23 (46.93)	0.462
Onset time of symptoms, mean (range), (weeks)	-	33.22 (16.29–38.29)	29.71 (10.00–35.14) ^#^	<0.05
Gestational weeks at time of ICP diagnosis mean (range), (weeks)	-	31.20 (10.14–39.57)	26.28 (9.00–39.43) ^#^	<0.001
Using UDCA drugs during pregnancy, mean (range), (weeks)	-	6.72(0.34–26.43)	9.89 (1.00–28.00) ^#^	<0.05
Using UDCA drugs during pregnancy, *n* (%)	-	59 (75.64)	42 (85.71)	0.173
<5 w, *n* (%)	-	28 (47.45)	13 (30.95)	0.098
≥5 w, *n* (%)	-	31 (52.54)	29 (69.05)	0.098
Peak level of TBA, (mean ± SD) (μmol/L)	-	24.63 ± 8.85	72.79 ± 44.91 **^#^**	<0.001
Peak level of TBA (before using UDCA drugs), (mean ± SD), (μmol/L)	-	19.50 ± 7.79	48.95 ± 45.97 ^#^	<0.001
Gestational age at peak level of TBA (before using UDCA drugs), mean (range), (weeks)	-	29.74 (10.14–40.71)	26.13 (9.00–39.43) ^#^	<0.05
Peak level of TBA (after using UDCA drugs), (mean ± SD), (μmol/L)	-	22.97 ± 9.54	66.11 ± 35.99 ^#^	<0.001
Gestational age at peak level of TBA (after using UDCA drugs), mean (range), (weeks)	-	34.72 (24.00–39.57)	33.09 (14.57–39.86)	0.193
Peak level of TBA (after delivery), (mean ± SD), (μmol/L)	-	9.20 ± 9.98	13.86 ± 14.07 ^#^	<0.05

The *p* values were adjusted by Bonferroni. * means compared to control group, *p* value < 0.05. ^#^ means compared to the mild ICP group, *p* value < 0.05. Abbreviations: BMI, body mass index; ICP, intrahepatic cholestasis of pregnancy; UDCA, ursodeoxycholic acid; TBA, total bile acid.

**Table 2:** Biochemistry function tests of pregnant women in the study.

Variables	At Diagnosis			*p* Value	At Delivery			*p* Value
	Control Group (*n* = 55)	The Mild ICP Group (*n* = 78)	The Severe ICP Group (*n* = 49)		Control Group (*n* = 55)	The Mild ICP Group (*n* = 78)	The Severe ICP Group (*n* = 49)	
PA (mg/L)	211 (194, 229)	197 (166, 219)	195 (140, 224)	0.051	215 (193, 236)	197 (165, 216) *	190 (154, 208) *	<0.05
TP (g/L)	61 (60, 63)	64 (61, 66)	63 (60, 65)	0.190	62 (59, 65)	63 (61, 66)	62 (59, 64)	0.095
Alb (g/L)	33 (31, 36)	34 (32, 36)	34 (31, 36)	0.887	34 (330, 38)	33 (32, 350) *	33 (31, 34) *	<0.05
GLB (g/L)	27 (25, 29)	30 (27, 31) *	28 (27, 31)	<0.05	28 (25, 30)	30 (28, 32) *	29 (27, 30)	<0.05
TBA (μmol/L)	2.5 (1.5, 3.6)	15.8 (12.4, 23.0) *	40.3 (22.2, 52.7) *^,#^	<0.001	2.4 (1.5, 3.6)	15.1 (10.0, 22.6) *	50.0 (36.9, 64.6) *^,#^	<0.001
TBIL (μmol/L)	7.5 (6.1, 9.6)	8.9 (6.8, 10.7)	9.2 (7.2, 11.7) *	<0.05	7.4 (5.9, 10.0)	8.7 (7.3, 10.0) *	8.7 (7.5, 10.3) *	<0.05
DBIL (μmol/L)	1.7 (1.4, 2.5)	2.1 (1.2, 2.9)	2.4 (1.5, 5.0)	0.053	1.7 (1.4, 2.7)	1.8 (1.3, 2.5)	1.9 (1.5, 3.3)	0.317
ALT (U/L)	13 (9, 16)	19 (12, 70) *	20 (11, 63) *	<0.001	12 (8, 16)	16 (10, 38) *	15 (10, 52) *	<0.05
AST (U/L)	17. (14, 20)	22 (16, 37) *	21 (16, 45) *	<0.001	16 (14, 20)	20 (16, 31) *	19 (16, 33) *	<0.001
ALP (U/L)	130 (103, 145)	181 (123, 223) *	148 (49, 220)	<0.05	150 (112, 197)	183 (140, 224) *	150 (116, 227)	<0.05
GGT (U/L)	14 (10, 20)	18 (10, 32)	16 (11, 53)	0.085	15 (11, 19)	19 (8, 30)	13 (8, 37)	0.414
LDH (U/L)	138 (132, 149)	172 (148, 207) *	167 (140, 177) *	<0.001	163 (147, 170)	175 (153, 196) *	171 (157, 208) *	<0.05
UREA (mmol/L)	2.92 (2.52, 3.37)	3.57 (2.92, 4.24) *	3.13 (2.66, 4.08)	<0.05	3.68 (3.09, 4.43)	4.13 (3.29, 4.87)	3.34 (2.89, 4.66)	0.250
UA (μmol/L)	156 (125, 185)	307 (248, 379) *	298 (233, 349) *	<0.001	317 (272, 362)	351 (297, 424) *	310 (27, 379) ^#^	<0.05
CYC (mg/L)	1.20 (1.01, 1.26)	1.18 (0.97, 1.52)	1.04 (0.85, 1.54)	0.357	1.28 (1.20, 1.40)	1.42 (1.13, 1.66)	1.24 (1.05, 1.54)	0.061

The *p* values were adjusted by Bonferroni. * means compared to control group, *p* value < 0.05. ^#^ means compared to the mild ICP group, *p* value < 0.05. Abbreviations: PA, pre-albumin; TP, total protein; Alb, albumin; GLB, globulin; TBA, total bile acid TBIL, total bilirubin; DBIL, direct bilirubin; ALT, alanine aminotransferase; AST, aspartate aminotransferase; ALP, alkaline phosphatase; GGT, gamma-glutamyl transpeptidase; LDH, lactate dehydrogenase; UREA, urea; UA, uric acid; CYC, cystatin C; ICP, intrahepatic cholestasis of pregnancy.

**Table 3:** Analysis of maternal complications and neonatal outcomes.

Variables	Control Group (*n* = 55)	The Mild ICP Group (*n* = 78)	The Severe ICP Group (*n* = 49)	*p* Value
Abortion, *n* (%)	0	0	1 (2.04)	0.207
Spontaneous preterm delivery, *n* (%)	3 (5.45)	0 (0)	3 (6.12)	0.097
Iatrogenic preterm delivery, *n* (%)	0 (0)	8 (10.26)	23 (46.94) *^,#^	<0.001
Fetal heart rate at delivery (median), (beats per minute)	140 (140, 142)	140 (140, 142)	141(140, 145)	0.067
PPH (mean ± SD), (mL)	258.9 ± 86.04	317.2 ± 127.8 *	350.0 ± 128.4 *	<0.001
24 h blood loss (mean ± SD), (mL)	365.2 ± 93.10	467.4 ± 140.3 *	494.2 ± 142.5 *	<0.001
Admission to NICU, *n* (%)	0 (0)	17 (21.79) *	26 (53.06) *^,#^	<0.001
Birth weight, mean (range), (g)	3179 (2390–3820)	3100 (1940–4450)	2845 (1420–4100) *^,#^	<0.05
Low birth weight, *n* (%)	3 (5.45)	4 (5.13)	8 (16.33) *^,#^	<0.05
Macrosomia, *n* (%)	0 (0)	4 (5.13)	1 (2.04)	0.194
Birth length, mean (range), (cm)	50 (46–53)	49 (44–54)	48 (37–51) *^,#^	<0.001
Apgar score < 7 at 5 min, *n* (%)	0 (0)	7 (8.97)	7 (14.29) *	<0.05
Meconium-stained amniotic fluid, *n* (%)	8 (14.55)	13 (16.67)	11 (22.45)	0.552
Adverse pregnancy outcomes, *n* (%)	10 (18.18)	30 (38.46) *	34 (69.39) *^,#^	<0.001

The *p* values were adjusted by Bonferroni. * means compared to control group, *p* value < 0.05. ^#^ means compared to the mild ICP group, *p* value < 0.05. Abbreviations: PPH, postpartum hemorrhage; NICU, neonatal intensive care unit; ICP, intrahepatic cholestasis of pregnancy.

**Table 4:** Analysis of maternal complications and neonatal outcomes in different groups.

Variables	Control Group (*n* = 15)	ICP with Non-Adverse Pregnancy Outcomes Group (*n* = 14)	ICP with Adverse Pregnancy Outcomes Group (*n* = 20)	*p* Value
Maternal age, mean (range), years	30.40 (22–39)	30.00 (25–38)	30.10 (20–37)	0.098
Maternal pre-pregnancy BMI, mean (range), (kg/m^2^)	21.30 (18.52–25.03)	20.95 (15.62–24.23)	19.97 (17.12–26.45)	0.087
Maternal BMI at delivery, mean (range), (kg/m^2^)	26.85 (22.72–33.87)	26.37 (21.88–29.30)	23.90 (19.98–26.84)	0.090
Weight gain in pregnancy, mean (range), (kg)	15.60 (3.50–25.50)	12.71 (7.50–20.00)	10.83 (5.50–20.00)	0.087
Multipara, *n* (%)	3 (21.43)	4 (28.57)	9 (45.00)	0.282
PA (mg/L)	215 (202, 236)	209 (180, 230)	218 (181, 234)	0.678
TP (g/L)	65 (63, 69)	65 (62, 68)	64 (61, 67)	0.576
Alb (g/L)	39 (38, 40)	40 (38, 41)	36 (33, 38) *^,#^	<0.05
GLB (g/L)	25 (25, 27)	26 (25, 28)	27 (26, 30) *	<0.05
TBA (μmol/L)	1.4(1.1, 2.2)	18.6 (11.2, 25.1) *	30.8 (19.3, 54.8) *^,#^	<0.001
TBIL (μmol/L)	5.8 (4.3, 7.9)	5.8 (4.9, 6.7)	11.2 (10.1, 15.1) *^,#^	<0.001
DBIL (μmol/L)	2.7 (2.0, 3.6)	3.0 (2.4, 4.1)	5.9 (5.1, 11.1) *^,#^	<0.001
ALT (U/L)	13 (11, 15)	15 (10, 39)	81 (46, 202) *^,#^	<0.001
AST (U/L)	17 (15, 18)	19 (14, 30)	48 (29, 106) *^,#^	<0.001
ALP (U/L)	74 (61, 138)	88 (57, 162)	192 (167, 246) *^,#^	<0.001
GGT (U/L)	12 (7, 16)	15 (10, 20)	260 (20, 46) *^,#^	<0.001
TBA > 40 μmol/L, *n* (%)	0 (0)	0 (0)	8 (40.00) *^,#^	<0.001
Gestational age at delivery, mean (range), (weeks)	39.76 (37.00–41.14)	38.68 (37.57–40.00)	35.79 (32.14–40.29) *^,#^	<0.001
Gestational age at delivery <37 wk, *n* (%)	0 (0)	0 (0)	16 (80.00) *^,#^	<0.001
Spontaneous preterm delivery, *n* (%)	0 (0)	0 (0)	2 (10.00)	0.102
Iatrogenic preterm delivery, *n* (%)	0 (0)	0 (0)	14 (70.00) *^,#^	<0.001
Cesarean delivery, *n* (%)	8 (57.14)	3 (21.43)	15 (75.00) ^#^	<0.05
Birth weight, mean (range), (g)	3174 (2600–3740)	3475 (3100–3965)	2616 (1420–3165) *^,#^	<0.001
Fetal heart rate at delivery (median), (beats per minute)	141 (140, 145)	140 (140, 142)	150 (146, 152)	0.132
Admission to NICU, *n* (%)	0 (0)	0 (0)	9 (35.00) *^,#^	<0.001
MSAF, meconium-stained amniotic fluid, *n* (%)	2 (14.29)	0 (0)	10 (50.00) *^,#^	<0.05
Apgar score < 7 at 5 min, *n* (%)	0 (0)	0 (0)	10 (50.00) *^,#^	<0.001
Gestational age at delivery <37 wk, *n* (%)	0 (0)	0 (0)	16 (80.00) *^,#^	<0.001
Spontaneous preterm delivery, *n* (%)	0 (0)	0 (0)	2 (10.00)	0.102
Iatrogenic preterm delivery, *n* (%)	0 (0)	0 (0)	14 (70.00) *^,#^	<0.001
Cesarean delivery, *n* (%)	8 (57.14)	3 (21.43)	15 (75.00) ^#^	<0.05

Data were shown as mean or median and interquartile range. The *p* values were adjusted by Bonferroni. * means compared to the control group, *p* value < 0.05. ^#^ means compared to ICP with non-adverse pregnancy outcomes group, *p* value < 0.05. Abbreviations: BMI, body mass index; PA, pre-albumin; TP, total protein; Alb, albumin; GLB, globulin; TBA, total bile acid; TBIL, total bilirubin; DBIL, direct bilirubin; ALT, alanine aminotransferase; AST, aspartate aminotransferase; ALP, alkaline phosphatase; GGT, gamma-glutamyl transpeptidase; NICU, neonatal intensive care unit; ICP, intrahepatic cholestasis of pregnancy.

**Table 5:** Concentrations of 38 urinary components of bile acids in different groups (nmol/mmol Cr).

Bile Acids	Control Group (*n* = 15)	ICP with Non-Adverse Pregnancy Outcomes Group (*n* = 14)	ICP with Adverse Pregnancy Outcomes Group (*n* = 20)	*p* Value
UDCA	0.17 (0.05, 0.43)	9.89 (1.72, 20.51) *	8.72 (3.11, 60.76) *	<0.001
HCA	0.67 (0.26, 3.60)	3.17 (0.91, 3.77)	2.42(0.54, 4.02)	0.619
CA	5.89 (3.04, 18.28)	32.24 (7.39, 110.1) *	36.64 (14.14, 96.07) *	<0.05
ω-MCA	9.06 (2.17, 15.69)	68.92 (46.23, 149.2) *	72.80 (16.44, 435.6) *	<0.001
α-MCA	0.04 (0.00, 0.15)	0.38 (0.08, 0.55)	0.36 (0.00, 1.58)	0.55
TUDCA	0.28 (0.13, 0.60)	2.09 (1.25, 5.28) *	31.56 (1.24, 92.22) *	<0.001
TCDCA	0.62 (0.39, 1.06)	1.57 (1.16, 2.98)	7.63 (0.81, 15.41) *	<0.05
TDCA	0.42 (0.28, 0.75)	0.88 (0.62, 2.84)	2.54 (0.79, 7.36) *	<0.05
THCA	2.88 (1.27, 4.81)	7.36 (3.64, 14.13)	60.33 (6.02, 110.4) *^,#^	<0.001
TCA	2.35 (1.40, 5.48)	12.58 (8.22, 25.19) *	81.75 (10.66, 254.7) *^,#^	<0.001
T-ω-MCA	9.08 (3.18, 15.42)	26.94 (19.78, 82.55)	206.4 (23.51, 376.3) *^,#^	<0.001
T-α-MCA	38.13 (14.90, 85.38)	78.97 (41.45, 192.7)	237.9 (33.27, 1054)	0.130
GUDCA	1.52 (0.60, 5.69)	35.68 (20.23, 75.87) *	399.1 (24.52, 1437) *	<0.001
GCDCA	0.78 (0.48, 4.00)	4.45 (3.04, 8.72) *	12.54 (4.19, 41.87) *	<0.001
GDCA	0.36 (0.17, 1.70)	3.80 (0.72, 10.07) *	2.94 (0.91, 21.89) *	<0.05
GHCA	4.56 (1.19, 7.11)	9.13 (4.46, 16.27)	47.51 (5.91, 168.8) *	<0.05
GCA	8.43 (4.40, 27.85)	54.39 (31.52, 151.0) *	363.3 (58.20, 1193) *	<0.001
UDCA-3-S	0.95 (0.52, 2.40)	362.7 (36.75, 1149) *	1304 (366.4, 4872) *	<0.001
CDCA-3-S	0.62 (0.32, 2.23)	3.48 (1.82, 6.01)	7.31 (2.27, 20.91) *	<0.05
DCA-3-S	1.15 (0.51, 2.42)	6.76 (2.24, 11.96) *	20.16 (3.72, 37.79) *	<0.05
LCA-3-S	4.85 (3.01, 5.42)	6.49 (4.75, 8.52)	15.89 (2.85, 254.9)	0.104
TCA-3-S	7.80 (4.03, 17.15)	21.59 (12.02, 29.85)	271.6 (31.00, 1391) *^,#^	<0.001
TUDCA-3-S	4.25 (1.87, 7.11)	381.4 (118.5, 954.5) *	9068 (251.4, 21569) *	<0.001
TCDCA-3-S	10.59 (3.14, 31.25)	26.08 (11.95, 59.92)	401.2 (45.24, 1181) *^,#^	<0.001
TDCA-3-S	15.02 (5.68, 64.89)	57.48 (32.53, 112.9)	340.0 (75.50, 1010) *^,#^	<0.05
TLCA-3-S	16.69 (4.67, 39.31)	52.36 (19.02, 95.64)	129.6 (26.63, 749.8)	0.065
GCA-3-S	6.43 (1.54, 11.40)	27.81 (20.63, 121.0)	525.8 (24.67, 2885) *	<0.001
GUDCA-3-S	19.55 (7.55, 45.34)	3529 (679.6, 9560) *	13,014 (1738, 107587) *	<0.001
GCDCA-3-S	32.98 (8.18, 88.78)	142.9 (74.35, 331.9)	1986 (206.2, 4007) *^,#^	<0.001
GDCA-3-S	45.79 (9.19, 146.2)	384.7 (105.4, 640.9)	1679 (357.6, 7676) *	<0.001
GLCA-3-S	30.39 (11.29, 42.60)	210.2 (55.34, 829.1) *	815.1 (88.08, 3810) *	<0.05
TCDCA-3-G	3.22 (1.35, 6.66)	8.54 (6.03, 17.14)	35.08 (5.43, 181.3) *	<0.05
TCA-3-G	1.85 (1.37, 7.40)	5.97 (2.88, 13.41)	31.48 (2.90, 118.8) *	<0.05
CDCA-3-G	1.84 (0.55, 9.05)	11.14 (2.11, 36.06)	12.87 (8.38, 46.72)	0.080
DCA-3-G	0.84 (0.24, 2.46)	3.91 (1.71, 7.40)	9.95 (3.73, 17.13) *^,#^	<0.001
GCDCA-3-G	22.94 (4.96, 46.12)	103.8 (38.76, 140.1) *	403.4 (47.91, 941.5) *	<0.05
GDCA-3-G	11.24 (5.70, 33.46)	49.67 (36.83, 85.15)	224.6 (46.09, 668.8) *^,#^	<0.001
LCA-3-G	0.14 (0.03, 1.33)	11.88 (0.61, 22.21) *	5.83 (2.10, 24.93) *	<0.05

Data are shown as median and interquartile range. The *p* values were adjusted by Bonferroni. * means compared to the control group, *p* value < 0.05. ^#^ means compared to ICP with non-adverse pregnancy outcomes group, *p* value < 0.05. Abbreviations: UDCA, ursodeoxycholic acid; HCA, hyocholic acid; CA, cholic acid; ω-MCA, ω-Muricholic acid; α-MCA, α-Muricholic acid; TUDCA, tauroursodeoxycholic acid; TCDCA, taurochenodeoxycholic acid; TDCA, taurodeoxycholic acid; THCA, taurohyocholic acid; TCA, taurocholic acid; T-ω-MCA, tauro-ω-muricholic acid; T-α-MCA, tauro-α-muricholic acid; GUDCA, glycoursodeoxycholic acid; GCDCA, glycochenodeoxycholic acid; GDCA, glycodeoxycholic acid; GHCA, glycohyocholic acid; GCA, glycolcholic acid; UDCA-3-S, ursodeoxycholicacid-3-sulfate; CDCA-3-S, chenodeoxycholic acid-3-sulfate; DCA-3-S, deoxycholic acid-3-sulfate; LCA-3-S, lithocholic acid-3-sulfate; TCA-3-S, taurocholic acid-3-sulfate; TUDCA-3-S, tauroursodeoxycholic acid-3-sulfate; TCDCA-3-S, taurochenodeoxycholic acid-3-sulfate; TDCA-3-S, tauro-deoxycholic acid-3-sulfate; TLCA-3-S, taurolithocholic acid-3-sulfate; GCA-3-S, glycolcholic acid-3-sulfate; GUDCA-3-S, glycoursodeoxycholic acid-3-sulfate; GCDCA-3-S, glycochenodeoxycholic acid-3-sulfate; GDCA-3-S, glycodeoxycholic acid-3-sulfate; GLCA-3-S, glycolithocholicacid-3-sulfate; TCDCA-3-G, taurochenodeoxycholic acid-3-glucuronide; TCA-3-G, taurocholic acid-3-glucuronide; CDCA-3-G, chenodeoxycholic acid-3-glucuronide; DCA-3-G, deoxycholic acid-3-glucuronide; GCDCA-3-G, glycochenodeoxycholic acid-3-glucuronide; GDCA-3-G, glycodeoxycholic acid-3-glucuronide; LCA-3-G, lithocholic acid-3-glucuronide; ICP, intrahepatic cholestasis of pregnancy.

**Table 6:** Diagnostic utility of urine biomarker levels in ICP with adverse pregnancy outcomes.

Biomarker (nmol/mmol Cr)	Training Set (*n* = 49)						Test Set (*n* = 14)
	AUC	*p* Value	95% CI	Sensitivity	Specificity	Cut Off Value	Specificity
THCA	0.755	<0.05	0.588–0.922	0.70	0.931	19.1	0.929
TCA	0.807	<0.05	0.679–0.935	0.70	0.862	27.8	0.786
T-ω-MCA	0.807	<0.05	0.677–0.937	0.65	0.931	115.8	0.929
TCA-3-S	0.848	<0.05	0.721–0.976	0.75	0.897	31.5	0.857
TCDCA-3-S	0.869	<0.05	0.768–0.970	0.80	0.828	41.4	0.714
TDCA-3-S	0.803	<0.05	0.664–0.943	0.65	0.966	176.8	1.000
GCDCA-3-S	0.860	<0.05	0.754–0.967	0.85	0.759	155.1	0.571
DCA-3-G	0.800	<0.05	0.675–0.925	0.55	0.931	9.27	0.929
GDCA-3-G	0.805	<0.05	0.671–0.940	0.55	1.000	193.3	0.929
Combination of the above biomarkers	0.886	<0.05	0.790–0.983	0.70	1.000	-	1.000
TBA (μmol/L)	-	-	-	0.37	0.367	10	0.214

Abbreviations: THCA, taurohyocholic acid; TCA, taurocholic acid; T-ω-MCA, tauro-ω-muricholic acid; TCA-3-S, taurocholic acid-3-sulfate; TCDCA-3-S, taurochenodeoxycholic acid-3-sulfate; TDCA-3-S, tauro-deoxycholic acid-3-sulfate; GCDCA-3-S, glycochenodeoxycholic acid-3-sulfate; DCA-3-G, deoxycholic acid-3-glucuronide; GDCA-3-G, glycodeoxycholic acid-3-glucuronide; TBA, total bile acid; AUC, area under the curve; CI, confidence interval.

**Table 7:** Correlation analysis between bile acid levels and clinical indices in the ICP with adverse pregnancy outcomes group.

Biomarker	TBIL		DBIL		ALT		AST		GGT	
	*r*	*p* Value	*r*	*p* Value	*r*	*p* Value	*r*	*p* Value	*r*	*p* Value
UDCA-3-S	0.366	<0.05	0.388	<0.05	0.532	<0.05	0.560	<0.05	0.369	<0.05
TCA-3-S	0.387	<0.05	0.360	<0.05	0.599	<0.05	0.617	<0.05	0.377	<0.05
TUDCA-3-S	0.382	<0.05	0.417	<0.05	0.587	<0.05	0.593	<0.05	0.461	<0.05
TCDCA-3-S	0.390	<0.05	0.342	<0.05	0.607	<0.05	0.629	<0.05	0.379	<0.05
GCA-3-S	0.376	<0.05	0.370	<0.05	0.497	<0.05	0.523	<0.05	0.354	<0.05
GUDCA-3-S	0.377	<0.05	0.410	<0.05	0.572	<0.05	0.588	<0.05	0.493	<0.05
GCDCA-3-S	0.415	<0.05	0.378	<0.05	0.611	<0.05	0.619	<0.05	0.413	<0.05

Abbreviations: UDCA-3-S, ursodeoxycholicacid-3-sulfate; TCA-3-S, taurocholic acid-3-sulfate; TUDCA-3-S, tauroursodeoxycholic acid-3-sulfate; TCDCA-3-S, taurochenodeoxycholic acid-3-sulfate; GCA-3-S, glycolcholic acid-3-sulfate; GUDCA-3-S, glycoursodeoxycholic acid-3-sulfate; GCDCA-3-S, glycochenodeoxycholic acid-3-sulfate; TBIL, total bilirubin; DBIL, direct bilirubin; ALT, alanine aminotransferase; AST, aspartate aminotransferase; GGT, gamma-glutamyl transpeptidase.

## Data Availability

Data produced in this study are presented in this paper.

## Abbreviations

ICP	Intrahepatic cholestasis of pregnancy
BAs	Bile acids
TBA	Total bile acid
APO	Adverse pregnancy outcomes
non-APO	Non-adverse pregnancy outcomes
UPLC-Triple TOF-MS	Ultra-high-performance liquid chromatography–triple quadrupole time-of-flight mass spectroscopy
UDCA	Ursodeoxycholic acid
THCA	Taurohyocholic acid
TCA	Taurocholic acid
T-ω-MCA	Tauro-ω- muricholic acid
TCA-3-S	Taurocholic acid-3-sulfate
TCDCA-3-S	Taurochenodeoxycholic acid-3-sulfate
TDCA-3-S	Tauro-deoxycholic acid-3-sulfate
GCDCA-3-S	Glycochenodeoxycholic acid-3-sulfate
DCA-3-G	Deoxycholic acid-3-glucuronide
GDCA-3-G	Glycodeoxycholic acid-3-glucuronide
UDCA-3-S	Ursodeoxycholic acid-3-sulfate
TUDCA-3-S	Tauroursodeoxycholic acid-3-sulfate
GCA-3-S	Glycolcholic acid-3-sulfate
GUDCA-3-S	Glycoursodeoxycholic acid-3-sulfate
MSAF	Meconium-saturated amniotic fluid
PTB	Preterm birth
NICU	Neonatal intensive care unit
ROC	Receiver operating characteristic curve
CA	Cholic acid
CDCA	Chenodeoxycholic acid
DCA	Deoxycholic acid
LCA	Lithocholic acid
HCA	Hyocholic acid
ω-MCA	ω-Muricholic acid
α-MCA	α-Muricholic acid
BMI	Body mass index
PA	Pre-albumin
TP	Total protein
Alb	Albumin
GLB	Globulin
TBIL	Total bilirubin
DBIL	Direct bilirubin
ALT	Alanine aminotransferase
AST	Aspartate aminotransferase
ALP	Alkaline phosphatase
GGT	Gamma-glutamyl transpeptidase
LDH	Lactatedehydrogenase
UREA	Urea
UA	Uric acid
CYC	Cystatin C
PPH	Postpartum hemorrhage
CR	Creatinine
